# Targeting protein folding in N‐Myc‐driven medulloblastoma

**DOI:** 10.1002/1878-0261.13395

**Published:** 2023-02-21

**Authors:** Gintvile Valinciute, Martine F. Roussel

**Affiliations:** ^1^ Department of Tumor Cell Biology St. Jude Children's Research Hospital Memphis TN USA

**Keywords:** chaperones, EIF4E, Hsp70, medulloblastoma, N‐Myc, protein translation

## Abstract

Selective targeting of N‐Myc‐driven Sonic hedgehog (SHH) medulloblastoma has been a challenge for many years and, despite decades of research, few targeted therapy opportunities exist. Recently, Kuzuoglu‐Ozturk et al. characterized the translatome of N‐Myc‐driven medulloblastoma as a promising therapeutic target. The study showed that N‐Myc controls a subset of members of the protein folding machinery that could be inhibited pharmacologically and validated a subset of Hsp70 functions as required for medulloblastoma progression *in vitro* and *in vivo*.

## Graphical abstract

A new study by Kuzuoglu‐Ozturk et al. identified a novel therapeutic vulnerability in N‐Myc‐driven Sonic hedgehog medulloblastoma. Selective translation of protein homeostasis pathways, including protein folding machinery components, drives tumour cell response to proteotoxic stress. Targeting the Hsp70 co‐chaperone leaves cells vulnerable to the proteotoxic stress induced by N‐Myc overexpression and inhibits N‐Myc‐driven medulloblastoma proliferation.

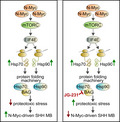

**Abbreviations**
BAG: Bcl‐2‐associated athanogene
BET: bromodomain and extra‐terminal
GEMM: genetically engineered mouse model
iPSC: induced pluripotent stem cells
MB: medulloblastoma
NES: neuroepithelial stem cells
SHH: Sonic hedgehog

The MYC protein family, including C‐Myc, N‐Myc and L‐Myc, are master regulators of multiple genes involved in core cellular processes and are overexpressed in numerous cancer entities, including paediatric malignancies [1, 2]. N‐Myc is historically associated with neuroblastoma [3], however, the gene is also overexpressed in a highly aggressive subtype of Sonic hedgehog (SHH) subgroup of medulloblastoma (MB) [4]. Due to its structure, direct targeting of the N‐Myc protein with small‐molecule inhibitors remains a challenge. Therefore, previous studies employed indirect targeting approaches, including the use of N‐Myc binding partners regulating N‐Myc stability (e.g. Aurora kinase A [5] or P300 [6]) or function (e.g. N‐Myc/MAX complex [7]). N‐Myc has also been targeted transcriptionally using small‐molecule inhibitors against bromodomain and extra‐terminal (BET) family proteins [8]. Finally, global inhibition of protein translation and metabolism via mTOR/PI3K/Akt axis proved useful in C‐Myc and N‐Myc overexpressed entities [9]. However, due to limited success of these approaches against N‐Myc‐driven paediatric cancers in clinical trials, new therapeutic strategies specific to N‐Myc are warranted.

A recent study by Kuzuoglu‐Ozturk et al. [10] unveiled a new therapeutic vulnerability in N‐Myc‐driven MB. They focused on the N‐Myc‐specific translatome and elucidated that, among others, N‐Myc regulates members of the protein folding pathway to stimulate SHH MB development. Authors used a previously developed N‐Myc‐driven SHH MB cell model GTML, derived from the germline genetically engineered mouse model (GEMM), where the human *MYCN* transgene was targeted to murine hindbrain neural progenitors [11]. Using genome‐wide ribosome profiling, they compared the translatome of GTML cells treated with two mTORC1 signalling inhibitors, rapalink‐1 and rapamycin. Rapalink‐1 inhibits both mTORC1 downstream effector pathways, S6 kinase and EIF4E, whereas rapamycin inhibits only S6 kinase‐mediated signalling. While rapamycin had a modest effect on translation efficiency, analysis of rapalink‐1‐treated cells revealed more than 350 genes selectively regulated at translational level. Interestingly, a large portion of those genes encoded proteins involved in protein homeostasis, including transport, oligomerization and folding. Due to the accelerated cell proliferation in aggressive cancers, pathways coping with proteotoxic stress (adverse effects due to damaged or misfolded proteins) are often activated [12]. Here, N‐Myc‐regulated specific translation of protein homeostasis machinery pointed to a group of potential previously overlooked targets in N‐Myc‐driven SHH MB. Remarkably, only 5% (12/262 proteins) of the protein folding machinery was translationally regulated in GTML cells, all of which were involved in distinct steps of Hsp70‐ and Hsp90‐mediated folding. Polysome profiling validated the selective translation of these proteins.

While Hsp90 inhibitors have previously shown some activity in MB [13], Hsp70 represents a new target in MB, and neither of the Hsp90 and Hsp70 chaperones has previously been shown to be specific targets in N‐Myc‐driven tumour cells. The authors compared multiple inhibitors targeting various parts of the Hsp90‐ and Hsp70‐driven protein folding machinery, including direct targeting of the ATPase activity of Hsp90 (NVP‐AUY922) and Hsp70 (VER‐155008) and targeting of two co‐chaperones of Hsp70, J‐domain proteins (MAL3–101) and Bcl‐2‐associated athanogene (BAG) proteins (JG‐231). Interestingly, only the latter showed more than 10‐fold selectivity for N‐Myc‐driven GTML cells compared to untransformed mouse embryonic fibroblasts. JG‐231 also prevented tumour growth *in vivo* emphasizing the translational potential of this target. However, these *in vivo* experiments were performed in flank tumours due to the inability of the assayed compound to cross the blood–brain barrier (BBB). Thus, better Hsp70 inhibitors that cross the BBB will need to be tested in the future to make targeting Hsp70 in medulloblastoma a valid therapeutic approach.

The mTOR/EIF4E axis has previously been shown to be strongly associated with SHH subgroup MB translation signalling [14]. Kuzuoglu‐Ozturk et al. unveiled that EIF4E signalling is essential for N‐Myc‐driven SHH MB. Encouraged by their translatome characterization results showing differences between rapalink‐1 and rapamycin treatment, the authors demonstrated that shRNA‐mediated EIF4E knockdown mimicked the pharmaceutical effect on the translation of protein folding machinery components. In order to better understand the connection between N‐Myc and EIF4E, the authors crossed their N‐Myc‐driven GTML GEMM with heterozygous EIF4E mice and observed a significant increase in animal survival. Thus, they concluded that EIF4E is required for N‐Myc‐driven tumour development. N‐Myc specificity was further demonstrated in additional models. By comparing human induced pluripotent stem cell (iPSC)‐derived neuroepithelial stem cells (NES) transformed by N‐Myc overexpression and NES cells from patients with Gorlin syndrome harbouring *PTCH1* mutations, they found that EIF4E strongly associated with SHH MB development. Both *in vitro* and *in vivo* treatment of NES cells with the BAG inhibitor, JG‐231, exhibited N‐Myc‐associated sensitivity to the inhibition of the BAG‐regulated subset of Hsp70 functions.

Taken together, Kuzuoglu‐Ozturk et al. for the first time not only characterized the translatome of N‐Myc‐driven SHH MB, but also suggested a new, previously overlooked therapeutic vulnerability in this paediatric brain tumour. While the authors elegantly described the potential of Hsp70 inhibition and its' association with N‐Myc function, this report raised several questions. While EIF4E signalling was found to be associated with SHH pathway signalling [14], it remains unclear whether the mechanistic interplay between SHH, N‐Myc and EIF4E could reveal other possible druggable proteins. Since N‐Myc drives multiple other cancers, would Hsp70 inhibition be applicable to all N‐Myc‐driven cancers? Will N‐Myc translational regulation of molecular chaperones including Hsp70 also be applicable to C‐Myc‐driven cancers? Finally, would inhibitors of Hsp70 specific to N‐Myc be applicable to C‐Myc‐driven tumours? The answers to these questions may help in determining the true clinical potential of Hsp70 in N‐Myc‐driven tumours.

## Conflict of interest

The authors declare no conflict of interest.
